# Assessing the Wider Implementation of the SHARP Principles: Increasing Physical Activity in Primary Physical Education

**DOI:** 10.3390/sports8010006

**Published:** 2020-01-09

**Authors:** Emma Powell, Lorayne Angela Woodfield, Alexander James Powell, Alan Michael Nevill

**Affiliations:** 1 Faculty of Education, Newman University, Birmingham B32 3NT, UK; 2 Faculty of Arts, Society and Professional Studies, Newman University, Birmingham B32 3NT, UK; l.a.woodfield@newman.ac.uk (L.A.W.); alexander.powell@newman.ac.uk (A.J.P.); 3 Faculty of Education, Health and Wellbeing, University of Wolverhampton, Wolverhampton WS1 3BD, UK; a.m.nevill@wlv.ac.uk

**Keywords:** primary physical education, physical activity, intervention, behaviour change theory, SHARP Principles

## Abstract

To assess the wider application of the SHARP (Stretching whilst moving, High repetition of skills, Accessibility, Reducing sitting and standing, and Promotion of physical activity) Principles intervention on children’s moderate to vigorous physical activity (MVPA) in physical education (PE), when applied by teachers and coaches. A quasi-experimental intervention was employed in nine primary schools (experimental, *n* = 6: control, *n* = 3) including teachers (*n* = 10), coaches (*n* = 4), and children (aged 5 to 11 years, *n* = 84) in the West Midlands, UK. Practitioners applied the SHARP Principles to PE lessons, guided by an innovative behaviour change model. The System for Observing Fitness and Instruction Time (SOFIT) was used to measure children’s MVPA in 111 lessons at pre- (*n* = 60) and post-intervention (*n* = 51). Seven interviews were conducted post-intervention to explore practitioners’ perceptions. Two-way ANOVA (Analysis of Variance) revealed that teachers increased children’s MVPA by 27.7%. No statistically significant change in children’s MVPA was observed when taught by the coaches. The qualitative results for teachers were ‘children’s engagement’, a ‘pedagogical paradigm shift’, and ‘relatedness’; and for coaches ‘organisational culture’ and ‘insufficient support and motivation’. The SHARP Principles intervention is the most effective teaching strategy at increasing MVPA in primary PE when taught by school based staff (rather than outsourced coaches), evidencing increases almost double that of any previously published study internationally and demonstrating the capacity to influence educational policy and practice internationally.

## 1. Introduction

Physical Education (PE) in the primary school setting is a unique subject, as it involves learning through being physical. Children learn how to move through physically performing skills [1] and children should be active for sustained periods of time in PE lessons [2]. The primary National Curriculum in England requires teachers to provide children with opportunities to become physically competent in a way that supports their health and fitness [2]. This implies that if children are given the chance to acquire a range of motor skills in PE then it may increase their likelihood of engaging in physical activities that would benefit their health, as children who are more proficient in fundamental movement patterns have an increased likelihood of participating in physical activities [3]. Furthermore, Public Health England (PHE) [4] reported that increasing the quality of PE classes can help to increase children’s PA. However, research into children’s PA levels in primary PE [5,6,7] repeatedly evidences that children are not meeting the recommended target of 50–80% MVPA [1]. If children are not active in PE, then they are not given the opportunity to develop their motor skills nor engage in PA of sufficient intensity and duration to benefit their fitness and health [8]. Hence, there is still a need to target low levels of PA in PE, which could be addressed through assessing the wider application of existing successful interventions that aim to increase children’s MVPA during primary PE lessons. 

In the wider implementation of interventions to increase children’s MVPA in primary PE it is important to consider the current context of the subject. For instance, the subject is often viewed as a low priority area amongst other curriculum subjects [9] and as a result, in England it is often contracted out to coaching companies [10]. Although many have expressed concerns over the use of sports coaches to deliver primary PE lessons [11,12], it is currently common practice for PE to be outsourced [13] and therefore it is important to assess the wider application of school-based interventions to the practice of both teachers and coaches. Additionally, school-based inspections have reported low levels of activity during PE lessons, irrespective of being taught by teachers or coaches [14], highlighting further the need to assess the delivery of both.To the authors’ knowledge, there is no research to date that has assessed the application of an intervention to increase children’s MVPA during PE lessons that evaluated the practice of both teachers and coaches. Furthermore, when considering the design of interventions that aim to increase children’s MVPA in PE lessons, it has been suggested that any behaviour change theory employed needs to target not only the individual teachers but also the community of practice in which they teach [7]. For school-based staff, this community of practice will be one school setting, however for sports coaches they could be working within several communities of practice such as a coaching company and a number of school settings. Therefore, this is something that will need to be considered when evaluating the practice of both teachers and coaches. 

It is also important to consider the design and effectiveness of previous interventions to increase children’s MVPA in PE, which tend to be split into two categories [15]: fitness-based [16,17,18,19] and teaching-based interventions [20,21,22]. The fitness-based interventions have evidenced the greater increases in children’s MVPA but it is the teaching strategy interventions that are of greater value as: (1) they align with the National Curriculum aims [2], and (2) not only will they increase children’s PA but they will also develop children’s motor skills and competence in a range of activities and sports. Previous teaching strategy interventions have produced a mean absolute increase of 6.27% in children’s MVPA during PE lessons, with intervention groups spending 14% more time in MVPA than control groups [15]. However, intervention evidence for increasing children’s MVPA in primary PE in England is limited [15], with only two studies conducting a PE specific intervention [23,24]. The intervention Motive8 was delivered by Motivate8 instructors, and it focused on both MVPA and children’s motor skills, producing absolute increases in MVPA by 12.4% [23]. More recently, the SHARP Principles intervention (Stretching whilst moving, High repetition of skills, Accessibility, Reducing sitting and standing, and Promotion of PA), a teaching strategy intervention grounded in behaviour change theory to target the teachers’ behaviours along with asking the teachers to focus on the SHARP Principles during the planning and delivery stages of their lessons [24], yielded a 30% absolute increase in children’s MVPA, almost double any other teaching strategy intervention. However, this intervention was only conducted with one intervention school and only assessed the practice of teachers, with the authors suggesting that further research was needed to test the efficacy of the SHARP intervention across a variety of school contexts and practitioners. Similar to the SHARP principles, a recent small-scale teaching strategy intervention in the US was also based on a set of teaching principles and evidenced absolute increases in both boys (5.8%) and girls’ (3.9%) MVPA [25]. The authors advocated that a strength of their study was the high-quality teaching principles that were integrated into the teachers’ practice [25]. Offering further support for teaching strategy interventions over fitness-based interventions. 

The use of teaching principles has been promoted by researchers to increase children’s active learning time in both PE lessons and extracurricular activities, for instance the ‘LETUS Play Principles’ [25,26], the SHARP Principles [24], and the SAAFE Principles [27]. All three sets of principles provide valuable advice on how to increase children’s PA during PE lessons/extra curriculum activities and aim to increase all children’s motor skills. However, it is the theory underpinning each set of principles that make the individual intervention strategies unique. For instance, the SAAFE Principles target children’s motivational climate within lessons and were informed by a number of motivational theories [27]. Whereas the LETUS PLAY Principles are embedded inside the PACES intervention, which also draws upon behaviour change theory, goal setting and Community-Based Participatory Research Principles (CBPR) [25]. The SHARP Principles are rooted in the SHARP Principles model, which is grounded in a unique combination of theoretical constructs including self-determination theory (SDT), elements from an ecological model, and key ingredients from the behaviour change taxonomy (BCT) [24]. The SHARP Principles model targets the teachers’ behaviour to create an active learning environment and embed the SHARP Principles in primary PE lessons, and is informed by previous research [7] in which children stated that they wanted to be moving and active in lessons, indicating that it is the teachers’ behaviour that needs to be changed in primary PE as they have ultimate control over the learning environment. What also makes the SHARP intervention unique is that the SHARP model targets the various levels of influence within English primary schools that may impact upon the teachers’ behaviour to create an active learning environment in primary PE lessons [24].

The SHARP Principles model has been previously shown to be effective on a small scale, using one control school and one intervention school, and it increased children’s MVPA in primary PE lessons by 30% [24]. Following on from the initial research, and taking into account the current climate of primary PE in England (i.e., PE being taught by both teachers and outsourced sports coaches), the next step, and therefore purpose of the current research, was to assess the wider implementation of the SHARP Principles intervention on children’s MVPA during primary PE when applied across several schools by teachers and sports coaches. The originality of this study is the innovative combination of theory underpinning the SHARP Principles that has been adapted from previous work [24] to target both teachers and sports coaches’ behaviour along with the levels of influence in both primary schools and coaching companies. Furthermore, to the authors’ knowledge, this study is the first teaching strategy intervention aiming to increase children’s MVPA in primary PE lessons that targets the practice of both primary school teachers and sports coaches. Therefore, this study has the potential to influence pedagogical knowledge and understanding in primary PE on an international scale.

## 2. Materials and Methods

### 2.1. Research Design, Participants, and Sampling Procedures

**Figure 1:**
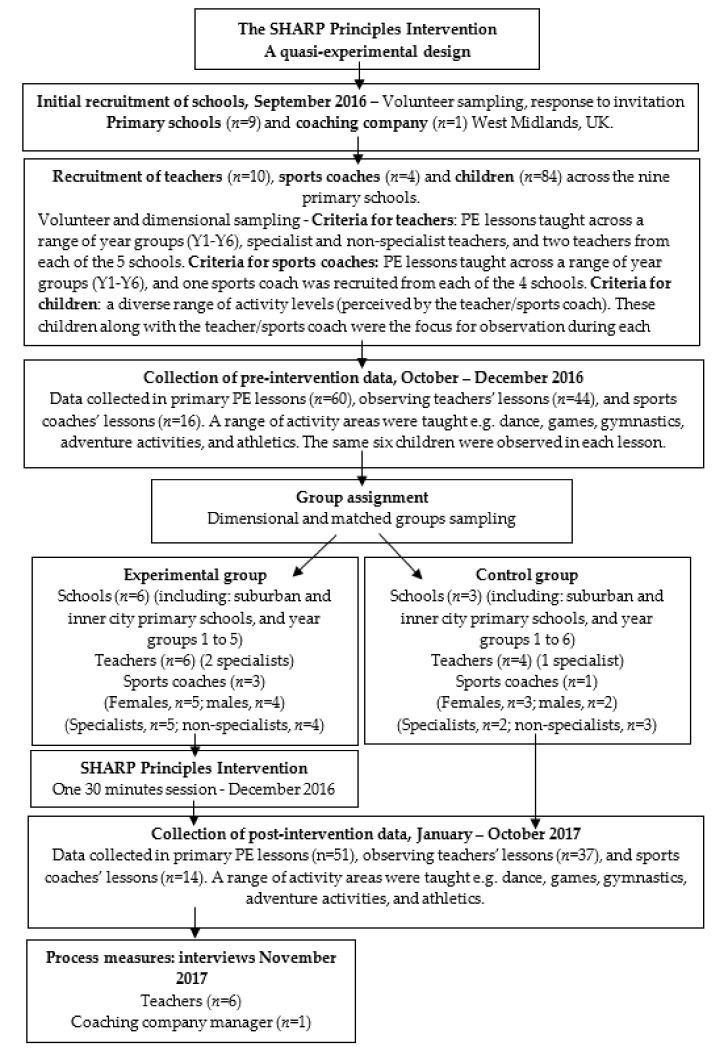
Flow chart representing research design and participant sampling procedures of the SHARP Principles intervention.

A quasi-experimental pre-test-post-test non-equivalent groups design [28] was employed, involving nine primary schools and one coaching company in the West Midlands, UK (Figure 1). Participants included: 10 primary school teachers (experimental, *n* = 6: control, *n* = 4), four sports coaches (experimental, *n* = 3, control, *n* = 1) and 84 children (aged 5 to 11 years). In September 2016, schools were selected via volunteer sampling, the first nine schools to reply to an invitation were recruited to the study; four of these schools had sports coaches delivering their PE lessons who were all employed by the same coaching company. Volunteer and dimensional sampling [28] was then applied to select teachers and coaches in each of the nine schools to represent diversity in the sample group. The criteria included: teachers and sports coaches, a range of year groups (Y1–Y6), and PE specialist and non-specialist teachers. In five of the primary schools, two teachers were recruited from each school, and in the remaining four schools, one sports coach was recruited in each school. Six children from each class taught by the participant teacher or sports coach were also selected through volunteer and dimensional sampling with the criteria of: equal number of males and females, and a diverse range of perceived (by the teacher/sports coach) activity levels. These children along with the teacher/sports coach were the focus for observation during each lesson. After collecting pre-intervention data, six teachers and three sports coaches were assigned to the experimental group and four teachers and one sports coach to the control group. Dimensional sampling was again applied at this stage, to ensure both the experimental and control groups contained a variety of characteristics (i.e., suburban and inner city schools, key stage one (aged 5–7 years) and key stage two classes (7–11 years), specialist and non-specialist teachers of PE, sports coaches, and male and female teachers); subsequently the experimental and control groups were then matched according to this diversity (Figure 1). The average class size was 30 (SD = 1).

Data were collected in 111 primary PE lessons (pre- *n* = 60; post-intervention *n* = 51). The experimental and control group teachers/sports coaches were observed teaching at least four PE lessons at both pre- and post-interventions stages of the research. Dimensional sampling was also applied to the lessons observed, ensuring that each teacher/sports coach taught a minimum of two different activity areas of the primary PE National Curriculum [2] (for instance, dance, gymnastics, games, athletics, and adventure activities) at pre-and post-intervention stages. All teachers who were in the experimental group and a coaching company manager were purposefully selected post-intervention to participate in individual semi-structured interviews. One of the coaching company managers was selected as the individual sports coaches in the experimental group had left the company before this final stage of the research. 

The study was reviewed and approved by the Research Ethics Committee at the lead researcher’s institution (Ethics application number S2015/030). Written informed consent was provided by the head teachers, coaching company director, a coaching company manager, teachers, sports coaches, and guardians of the children involved. Additionally, verbal consent was sought from the children.

### 2.2. Procedures 

#### 2.2.1. The SHARP Principles Intervention

**Figure 2:**
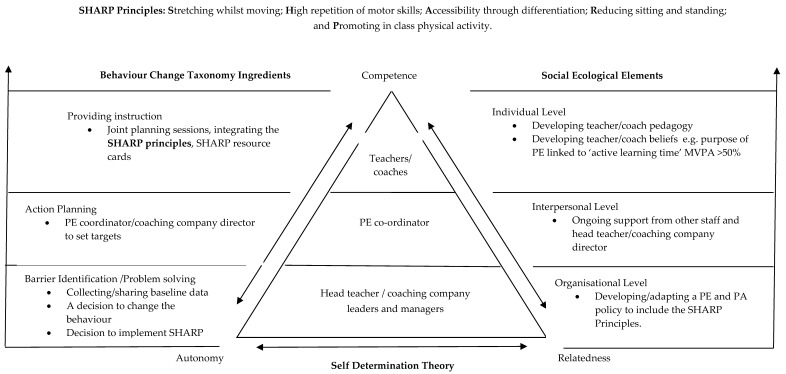
The SHARP Principles model: designed to increase children’s active learning time during primary PE lessons (adapted from [24]).

The SHARP Principles intervention is grounded in the SHARP Principles model (Figure 2). This model was designed to target teachers’ behaviour to increase the time children spend in MVPA during primary PE lessons. The SHARP Principles intervention was initially piloted using only teachers [24], however this study has targeted both teachers and sports coaches, therefore the SHARP model (Figure 2) and theoretical constructs (Table 1) have been adapted from their initial use to account for this. The SHARP Principles (Table 2) are a set of pedagogical guidelines for practitioners to consider during the planning and delivery stages of their PE lessons. They are not affected by curriculum, activity type, or teaching style. The SHARP Principles have been informed by a previous exploratory study [7], which drew upon observations of teachers’ and children’s behaviour during primary PE lessons and their perceptions of active learning time during lessons. 

The SHARP Model contains a unique set of theoretical constructs to target the teachers’ or sports coaches’ behaviours during primary PE lessons. The aim is for the practitioners to implement the SHARP Principles in PE lessons which will result in children engaging in more MVPA and having increased opportunities to develop their motor skills. The SHARP Model targets the key members of staff in a primary school setting and/or coaching company. This is represented at a triangular level, i.e., head teacher/coaching company leaders and managers, at the base of the triangle, as it is predicted that without their support the intervention would not be successful. At the second level of the triangle is the intervention lead in the school i.e., the PE coordinator (or this may be an appointed member of staff in the school/coaching company), followed by the individual teachers/sports coaches at the top of the triangle. To interlink these roles, SDT (Self-Determination Theory) [29] was applied to motivate the teachers/sports coaches to change their behaviour, through a supportive yet autonomous role with the premise of developing their competency to embed the SHARP Principles into their PE lessons. Working alongside SDT were three elements of an ecological model [31] including the individual, interpersonal, and organisational levels. At the organisational level, the school/coaching company were asked to adapt their PE/PA policy to include a statement regarding teachers/sports coaches implementing the SHARP Principles into their PE lessons. At the interpersonal level, ongoing support was encouraged from the head teacher, intervention lead/coaching company leaders and managers, and other staff members in the use of the SHARP Principles during PE lessons. The individual level targeted teachers’/sports coaches’ ‘knowledge and beliefs’ in terms of primary PE, with a target of >50% MVPA being an aim of their lessons. Furthermore, their ‘pedagogical knowledge’ was targeted through the use of the SHARP Principles and the related resource cards. In addition to SDT and elements from an ecological model, three active ingredients of the BCT (Behaviour Change Taxonomy) [30] were implemented. These were ‘barrier identification’, ‘action planning’, and ‘providing instruction’ (e.g., the SHARP Principles).

The input from the researchers involved meeting with the headteachers/coaching company leaders and managers, and/or lead for the intervention for an introductory meeting. Following this, the researchers met with the teachers/sports coaches and explained the SHARP Principles and SHARP resource cards; these meetings lasted approximately 30 min.

#### 2.2.2. Physical Activity Measure: System for Observing Fitness and Instruction Time (SOFIT)

The System for Observing Fitness and Instruction Time (SOFIT) is a systematic observation instrument that collects data across the variables of ‘children’s PA levels’ (lying down, sitting, standing, walking/moderate, and vigorous), ‘lesson context’ (management, knowledge, fitness, skills, games, and other), and ‘teacher interactions’ (in-class promotion of PA, out of class promotion of PA, and no promotion of PA) [38]. SOFIT is considered a valid and reliable instrument in the objective assessment of PE lessons [39], as the PA variables have been validated against accelerometers, with statistically significant positive correlations (*r* = 0.67; *P* < 0.01) [40], along with heart rate monitors [41] and pedometers [42]. SOFIT was used as the primary objective measure to assess children’s MVPA at pre- and post-intervention. A total of 111 PE lessons were observed (pre- *n* = 60 and post-intervention *n* = 51). Data were collected by trained observers (*n* = 6) and training included: watching SOFIT training videos, becoming familiar with the SOFIT protocols [38], and field practice. Inter-observer reliability ratings were set at both pre- and post-intervention data collection points and two in field reliability checks took place. Training time ranged from 5–20 h and all inter-reliability checks were above 89% in each SOFIT variable. 

Six children were observed during each PE lesson on a rotational basis. Six children in each observed class were selected via dimensional sampling with the criteria being a range of activity levels in the sample. This was to help maintain consistency across the pre- and post-intervention data. The SOFIT observation instrument is a class level measure, and therefore provides a mean % of activity for the six observed children for each observed lesson. A pacer was used on an MP3 player to pace the time sampling intervals in which observers were prompted to observe for a 10 second period followed by a 10 second period to record observations [38]. Observations began once 51% of the class had entered the working area and observations ended once 51% of the class had left the working area (38). All observers positioned themselves on the edge of the working area, to reduce any observer reactivity. Full details of the SOFIT procedures can be found elsewhere [38].

#### 2.2.3. Process Measure: Semi-Structured Interviews

Semi-structured interviews (*n* = 7) were conducted post-intervention to explore practitioners’ perceptions and experiences of taking part in the intervention. This sample included all teachers in the experimental group (*n* = 6) and the Learning and Development Manager of the coaching company, as the individual sports coaches in the experimental group had left the company before this final stage of the research. Qualitative data has been stated as a useful approach to employ alongside objective measures to assess the process measures of school-based PA interventions [43]. The interviews were a particularly beneficial method to investigate the features and levels of the SHARP Principles model. The interview questions mirrored the components of the interventions, e.g., ‘Do you feel as though your practice has changed/or not as a result of the intervention?’ and ‘Could you share with me any facilitators or barriers to implementing the SHARP Principles during your physical education lessons?’ A dictaphone was used to record the interviews, and data were collected, transcribed, and analyzed by two researchers.

The overall trustworthiness of the interview data was increased due to the integration of the following four concepts: credibility, transferability, dependability, and confirmability [44]. The notion of credibility was addressed by member checking during the interviews through clarification of answers and meanings in the response to interview questions. Furthermore, credibility was also enhanced through critical reflections post interview between the research team, and the systematic approach adopted along with verbatim extracts included in the write up of the study. The transferability of the research is sought through the contextual information provided of the context understudy and the clear boundaries of the study’s scope. The concept of dependability was enhanced through the description of the methods employed and overall research design. Finally, bracketing of researchers’ assumptions took place throughout the study to enhance the notion of confirmability.

### 2.3. Data Analysis

#### 2.3.1. Quantitative Analysis

Due to the underlying differences in the training and support networks of teachers and sports coaches, it was decided to conduct separate data analysis for these two groups; consequently for each of the two data sets (data set 1 = teachers; data set 2 = sports coaches) the following statistical steps were applied. 

In the first instance, as the SOFIT instrument is a class level measure, the mean percentages of the SOFIT categories (dependent variables) were calculated for each observed lesson (teachers: pre- (*n* = 44), post-intervention (*n* = 37); coaches: pre- (*n* = 16), post-intervention (*n* = 14)). The total means and related descriptives were then determined for each data set, and following this, a two-way ANOVAwas conducted. A two-way ANOVA was selected as it enabled the researchers to measure the effect of two independent variables on one dependent variable [45]. Accordingly, the effect of the independent variables (fixed factors) of ‘group’ (i.e., intervention and control) and ‘time’ (pre- and post-intervention) were measured against the dependent variable of ‘MVPA’. Partial eta squared (η_p_^2^) (small (0.01), medium (0.06), and large (0.14)) [46], was used to interpret the interaction effect sizes for changes in pre- and post-intervention data in each of the data sets (i.e., teachers and sports coaches). Statistical analyses were conducted using the Statistical Package for the Social Sciences v.26, and the alpha level was set at *P* < 0.05. Statistical assumptions for a two-way ANOVA were adhered to [28], including checking for homogeneity of variances and normality of residuals.

#### 2.3.2. Qualitative Analysis

Interpretative phenomenological analysis (IPA) [47] was used to analyse the interview data. IPA was selected as it acknowledged the hermeneutic nature of the research team in relation to their understanding and values of primary PE (i.e., over 10 years’ experience of teaching in primary PE and coach education). IPA is a version of phenomenology which accepts that the data will always be affected by the researchers’ views and hence their interpretation of the data [48]. An IPA approach is grounded in the three key concepts of phenomenology (human experience), hermeneutics (interpretive nature), and idiography (detailed examination) [47]. The first step of the analysis involved reading and re-reading the transcripts, with initial notes being made in order to capture any initial impressions. The second step involved the creation of exploratory comments which were separated according to: descriptive (description of the content), linguistic (specific use of language), and conceptual (interrogation and interpretation) comments. The third step of the data analysis involved data reduction to create emergent themes, this involved breaking up the narrative and fragmenting the hermeneutic cycle. This whole process of data analysis was repeated for each transcript. The final steps involved the abstraction of themes from each transcript, at this point the themes were drawn together and a structure was produced providing organisation to the analysis. The main themes were then compared against the SHARP Principles model to identify the most effective components in the model.

## 3. Results

### 3.1. Physical Activity Measure (SOFIT)

#### 3.1.1. Teachers’ Results 

**Figure 3:**
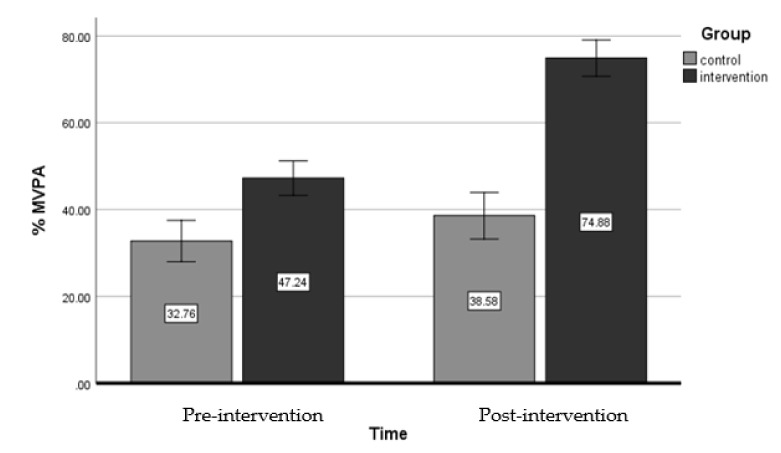
Mean % of MVPA during primary PE lessons when taught by teachers in both intervention and control groups at pre- and post-intervention.

A large significant interaction effect between the independent variables (fixed factors) of ‘group’ (i.e., intervention and control) and ‘time’ (pre- and post-intervention) on the dependent variable of ‘MVPA’ was evident in the teachers’ data, (*F* (3,81) = 11.07, *p* = 0.003, η_p_^2^ = 0.316). This indicates that the intervention had a significant effect on %MVPA in the teachers’ primary PE lessons post-intervention (Figure 3). Specifically, the teachers’ MVPA data in the intervention group increased by a mean of 27.64% from pre- (M = 47.24, SD = 11.52) to post-intervention (M = 74.88, SD = 6.64) data collection points. Whereas, the teachers’ MVPA data in the control group stayed relatively stable between pre- (M = 32.76, SD = 10.46) to post-intervention (M = 38.58, SD = 11.55) conditions. 

#### 3.1.2. Sports Coaches’ Results

There was no significant interaction effect between the independent variables (fixed factors) of ‘group’ (i.e., intervention and control) and ‘time’ (pre- and post-intervention) on the dependent variable of ‘MVPA’ in the sports coaches’ data, (*F* (3,30) = 0.12, *p* = 0.74, η_p_^2^ = 0.004). This indicates that the intervention did not have any significant effect on the %MVPA in the sports coaches’ primary PE lessons post-intervention. Specifically, the sports coaches’ %MVPA data in the intervention group stayed stable for both pre- (M = 40.94, SD = 16.40) and post-intervention (M = 38.04, SD = 10.74) conditions, as did the sports coaches’ data for the control group (pre- *M* = 53.08, SD = 19.68; post- M = 45.98, SD = 14.37).

### 3.2. Process Measure (Semi-Structured Interviews)

**Figure 4:**
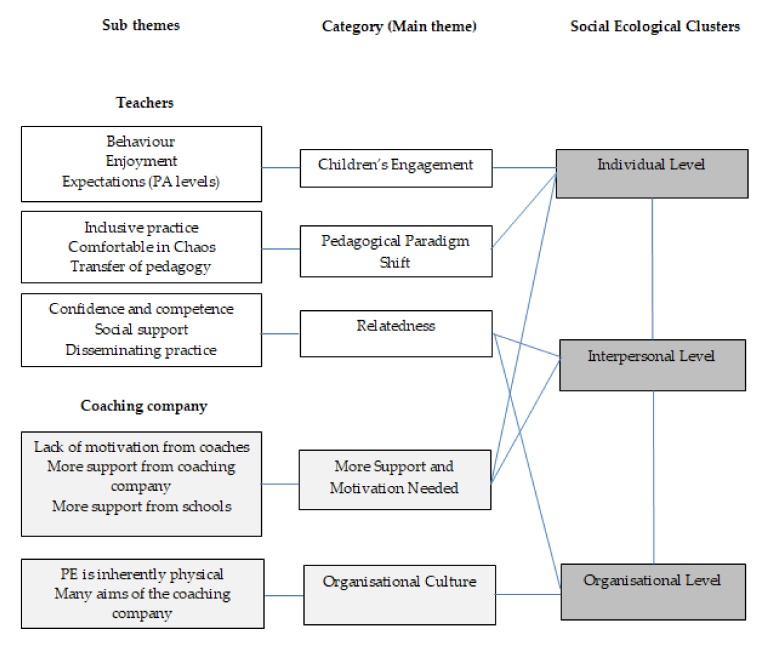
Teachers’ and coaching company’s perspectives of participating in the SHARP Principles intervention: a representation of sub themes, categories, and social ecological clusters.

The main qualitative themes and related sub themes from the teacher interviews were ‘children’s engagement’ (behaviour, enjoyment, and expectations), a ‘pedagogical paradigm shift’ (inclusive practice, comfortable in chaos, and transfer of pedagogy), and ‘relatedness’ (confidence and competence, social support, and disseminating practice). The main themes and sub themes from the interview with the coaching company manager were: ‘more support and motivation needed’ (lack of motivation from coaches, more support from coaching company, and more support from schools), and ‘organisational culture’ (PE is inherently physical, and the many aims of the coaching company). The categories from each data set were then matched to related social ecological layers from within the SHARP Principles model (Figure 4).

#### 3.2.1. Teachers’ Process Measures Results

##### Category: Children’s Engagement

It was evident from the teachers’ interview data that the SHARP Principles intervention had impacted positively upon the ‘children’s engagement’ during PE lessons. The sub themes that were prominent in this category were: behaviour, enjoyment, and expectations. The teachers reported that as a result of the intervention they had noticed an improvement in the children’s behaviour during PE. A common thread from the teachers was that as the children were more active and focused in lessons; this resulted in fewer opportunities for children to misbehave. Furthermore, the teachers’ reported that the children enjoyed their PE lessons that were focused on the SHARP Principles, and the children’s expectations of how active they would be in PE lessons grew each week as they became accustomed to the change in activity levels. 

For instance:

“I think that the good thing about the SHARP Principles was that there were less issues, as there was less time for the children to be messing around and causing problems because you are keeping them active all the time.”

“I think all the children were really positive about it.”

“I think the not sitting around for me was the biggest change, I think it was the hardest one to do at the start but the children actually liked being more active but it was interesting because they said they were tired, they needed a drink and as the weeks have gone on that started to slow down so I think their stamina has increased and they have got used to the activity as we have gone along.”

##### Category: Pedagogical Paradigm Shift

The teachers’ interview data also highlighted the impact the SHARP Principles intervention had on their PE pedagogy. The four emerging sub themes within the ‘pedagogical paradigm shift’ category were: inclusive practice, comfortable in chaos, transfer of pedagogy, and breaking the cycle. In relation to ‘inclusive practice’, the teachers reported that as a result of the intervention they are now more aware of every child in PE lessons and what they are doing e.g., on task and active. In addition, teachers discussed how they became more ‘comfortable in chaos’ during PE lessons, in the sense that a PE lesson does not have to look neat and tidy to be effective. Teachers became comfortable with every child having a piece of equipment and every child being active at the same time. It was also highlighted during the interviews that teachers began to transfer their pedagogical skills from other curriculum subjects to PE lessons. For example, teachers expressed that they would not have children queuing for their turn in Maths and English lessons nor would they stop the whole class if one child did not understand the task, so they began to transfer this practice over to PE lessons as a result of the intervention. The sub theme of ‘breaking the cycle’ encompasses teachers’ awareness of their change in pedagogy as a result of the intervention. Teachers discussed that the intervention gave them a ‘new look’ for PE, approaching lessons in a more creative way, breaking free from their habitual practice of sedentary PE lessons. Examples of teachers’ comments included:

“I’m more conscious that the children are on task and doing something, so no one is really off task not taking part in the skill or the activity, I ensure that everyone is enjoying it and having fun and that there is a high level of activity.”

“It’s trying to get yourself out of the habit of doing it one way, it’s just shaking off what you used to do but once you carry on with it, it just becomes easy. It’s accepting that even if it does look quite chaotic you’ve just got to let it go and let them have a go rather than stopping everyone and saying watch me… I’m getting more comfortable in chaos now… I suppose you have to recognise when it is controlled chaos and not just chaos.”

“In Maths and English, if someone is unable to do something you would not stop the whole class from working, teachers have those skills so it’s just transferring them to PE lessons.”

“I think it gave me a new look for PE, a way of thinking creatively about getting them active rather than getting them all just to run around. It was relatable to the learning objective, it is about being fit and healthy but also the skills… you had to think, how can I develop that skill whilst keeping them active.”

##### Category: Relatedness

The final main category from the teachers’ interview data was ‘relatedness’, with the emergent sub themes of: competence and confidence, social support, and disseminating practice. It was evident from the interview data that the SHARP Principles gave the teachers the confidence and knowledge to deliver active and purposeful PE lessons. The teachers expressed that they see themselves as being a better teacher in PE lessons, teaching in a completely different way and they felt that the SHARP Principles, although flexible, gave them a structure to follow. The teachers also discussed the importance of social support, being able to discuss their ideas for PE lessons with someone else. Having the support of the head teacher was also important, supporting their new way of teaching in PE lessons. In addition, all intervention teachers stated that they would like to share with the rest of the school their new knowledge and way of teaching PE lessons. Some teachers expressed that there was now a visible gap in their teaching of PE and other staff in the school. Teachers expressed concern over practice they frequently observed in other teachers PE lessons, with children sitting and standing for long periods of time:

“I feel as though I’m a much better teacher of PE than I was, it was something that I wasn’t very confident in and now I do feel as though I have an ingrained structure that I can follow.”

“It is good to talk to somebody else about it because that helps, if you have someone in your school who is really PE focused you can talk to them and go through the cards and maybe they can give you ideas.”

“I think the head wants us to close the gap now between us and the rest of the teachers.”

“I think it’s something we need to get across the school, as we walk through lessons and we see that teachers have the children all sat down and that can cause behaviour issues as well rather than just stopping one or two and letting the others carry on.”

#### 3.2.2. Coaching Company’s Process Measures Results

##### Category: Insufficient Support and Motivation

One of the main themes from the interview data with one of the coaching company’s managers was ‘more support and motivation needed’, and within this main category were the sub themes of: lack of motivation from coaches, more support from the coaching company, and more support from schools. The manager highlighted that the coaches may not have been motivated to implement the SHARP Principles into their PE lessons. The reasons for this lack of motivation were attributed to the individual coaches in the intervention group wanting to change jobs and move on from the coaching company. The interview data also highlighted that the coaching company could have given more support to the intervention coaches, as the manager stated that they could have done a lot more as a company to support the coaches. Furthermore, the data highlighted that the coaches did not receive any support from the schools either in implementing the SHARP Principles in their PE lessons, thus indicating that the intervention coaches did not receive sufficient support from the coaching company nor the schools in which they were teaching:

“Maybe it was the coaches’ motivation of not wanting to implement the SHARP Principles, so their motivation levels may have been a barrier. I think the three coaches were looking to see what their next move was, away from the company, which could have made an impact.”

“I think from the management side of the company we could have given a bit more support with it to the coaches.”

“Some schools were more interested than others and a couple asked how it went, I think they become interested in it after they get good results.”

##### Category: Organisational Culture

The second main theme from the coaching company interview data was ‘organisational culture’ and the associated sub themes were: PE is inherently physical, and the many aims of the coaching company. From the interview data, the manager discussed how the company, and consequently the coaches, assume that PE lessons are physical in nature and hence the coaches may have considered their existing teaching of PE effective, without the need for intervention. The interview data also highlighted that the coaching company had many different aims that they sought their coaches to achieve during primary PE lessons. For instance, they wanted PE lessons to be active, social, develop leadership skills, and sport skills. Being active in PE lessons appeared to be a taken-for-granted concept, a by-product of practicing skills:

“I think because of the name physical education, there has to be a high level of movement to fit in with what they are learning… maybe they didn’t see it as being valuable because they already knew what to do and felt like they didn’t need it.”

“We think there are lots of different aims, so the main one being to develop the children and then within that there are loads of different skills, so physical skills, sport specific skills, obviously the health and well-being side, social side, and holistic side.”

## 4. Discussion

The main aim of this study was to assess the wider implementation of the SHARP Principles intervention on children’s MVPA during PE, when applied by teachers and sports coaches. To the authors’ knowledge this is the first teaching intervention study to target both teachers’ and coaches’ behaviour to increase children’s MVPA during primary PE lessons. Furthermore, the originality of this study is the innovative combination of theory underpinning the SHARP Principles that has been adapted from previous work [24] to target both teachers’ and sports coaches’ behaviour, along with the levels of influence in both primary schools and coaching companies. The results indicated that the SHARP Principles intervention had a statistically significant effect on children’s MVPA in primary PE lessons when taught by the teachers in the intervention group, and yielded an absolute increase of 27.4% to a mean average of 74.88% MVPA in PE lessons. However, the results also indicated no statistically significant change in children’s MVPA% when taught by sports coaches in the intervention group. Thus, the SHARP Principles intervention was effective when applied by teachers but had no effect when applied by sports coaches. The qualitative findings suggested that the organisational culture and insufficient support in the coaching company and contracted schools impacted upon the sports coaches’ motivation to implement the SHARP Principles in their PE lessons. Whereas the qualitative findings highlighted that the teachers were motivated to implement the SHARP Principles from developing a sense of ‘relatedness’ with other teachers in the school which included the support from the head teacher. Teachers also advocated that the SHARP Principles intervention had a positive impact on both the children’s engagement in PE lessons and their pedagogical practice.

The first small scale study of the SHARP Principles intervention [24], produced a 30% absolute increase in children’s MVPA during primary PE. When implemented on a broader scale and applied across a range of schools and year groups, the current study provides further evidence that the SHARP Principles intervention is effective when used by teachers, as a similar absolute increase of 27.4% was produced from pre- to post-intervention. Additionally, the average mean MVPA% (74.9%) in the teachers’ lessons met and exceeded the recommended target of >50% MVPA during PE lessons [1], which encompassed a range of lesson content including games, dance, and gymnastic activities. When placing this result against previous published teaching strategy intervention studies, the SHARP Principles intervention evidences absolute increases in children’s MVPA% almost double that of any previous teaching strategy intervention internationally [15]. Additionally, when making comparisons with other interventions [26] based on a set of ‘principles’ to increase children’s PA in PE and extracurricular sessions, it can be argued that the SHARP Principles is the most effective intervention in primary PE, which may be attributed to the underlying theoretical constructs that the SHARP Principles are embedded within. Therefore, it is important to discuss the combination of theoretical constructs applied to the intervention and why behaviour changes and positive results were seen in the teachers’ lessons but not the coaches’ lessons. Considering the commonplace use of outsourced sports coaches in primary schools, these results could have implications for future practice in primary PE in England and need to be analysed further. Thus, the behaviour change constructs will be critically examined according to the social ecological clusters that are situated in the SHARP Principles theoretical model.

### 4.1. Individual Cluster

The implementation of the SHARP Principles intervention involved targeting the teachers’ and coaches’ individual behaviour during primary PE lessons. This decision to target the instructors’ behaviour rather than the children’s behaviour was informed by prior research [7] which highlighted that children wanted to be moving during PE lessons and it was the instructors who had the ‘power’ to enable this to happen or not. Thus, part of the intervention involved developing the teachers’ and sports coaches’ knowledge of the SHARP Principles which would provide them with strategies to implement during lessons in order to create an active learning environment. However, both the quantitative and qualitative findings indicated that the teachers successfully employed the SHARP Principles into their lessons, whereas the sports coaches did not. This provides further evidence that having the knowledge of ‘how’ to increase active learning time (e.g., through a set of guiding principles) during PE lessons is often not enough and behaviour change theory is required to target individuals and communities of practice [7].

For the teachers, the qualitative data evidenced that there was a pedagogical paradigm shift in their practice. Teachers expressed that they became more confident and the intervention gave them a ‘new look’ for PE lessons. The teachers began to reflect upon how they previously taught PE, questioning why they had taught in such a sedentary way; as has been repeatedly evidenced in previous literature [5,7,15]. Furthermore, teachers reported children were more active and focused in PE lessons which resulted in better behaviour. This positive outcome would have given the teachers a sense of confidence in their change of practice and research suggests that if a teacher is not happy with their current teaching practices, there may be a desire to change [49]. Primary school teachers generally have low confidence in their ability to teach primary PE lessons [7], however from this research it appears that the SHARP Principles intervention provided a platform for the teachers to change their practice, as research indicates that if a teacher gains access to powerful strategies through effective learning opportunities, then the teacher has the means and support to make changes [49].

In contrast, the qualitative data highlighted that the sports coaches lacked motivation to implement the SHARP Principles into their PE lessons. In relation to SDT, if one of the components is missing (i.e., competence, autonomy, relatedness) then an individual may not be motivated to complete a given task [29]. The sports coaches had the same input as the teachers in terms of gaining knowledge of the SHARP Principles (competence), however as the intervention evidenced no effect on children’s MVPA when taught by the sports coaches, it would lead us to analyse other components of the intervention. The coaches also had the same autonomy as the teachers in regards to having the choice of lesson content to deliver, with the SHARP Principles added as a layer to their lessons. Subsequently, the component of SDT that appears to have differed between the two groups was ‘relatedness’. The coaching company manager stated that the sports coaches in the intervention group were considering their next move during the intervention and hence they would be soon leaving their post as sports coaches teaching PE in primary schools. This could have led to a lack of ‘relatedness’ between themselves and the coaching company as well as the individual schools and classes in which they were teaching. This could help to explain their lack of motivation to implement the SHARP Principles during their PE lessons. Thus, if schools are going to outsource their PE lessons to sports coaches then both the contracted school and the coaching company need to consider professional development opportunities for the sports coaches, which could help to motivate the individual coaches.

### 4.2. Interpersonal Cluster

Interpersonal relationships between the networks in which people work are important influences upon their behaviour [31]. For the teachers, the interpersonal relationships would primarily be bound within one work setting, however, for the sports coaches their interpersonal relationships would be spread across multiple settings and social networks. For instance, the sport coaches had interpersonal relationships with groups in the coaching company as well as the primary schools in which they taught PE. The qualitative data suggested that the teachers and sports coaches had contrasting experiences in relation to the social support they received during the intervention. For example, the teachers commented how important it was to have the support of the head teacher during the intervention along with support from other teachers within the school setting. Whereas the sports coaches appeared to have limited support from either of their social networks, and therefore needed more support than that provided by the coaching company and the school settings in which they worked. Again this issue of support from social networks can be linked to the concept of ‘relatedness’ within SDT [29]; if the coaches did not feel supported during the intervention then this would have impacted upon their feelings of ‘relatedness’ and consequently their motivation to implement the SHARP Principles into their PE lessons. The findings related to the interpersonal layer would imply that consideration needs to be given to how sports coaches can be supported by both the contracted school and the coaching company.

### 4.3. Organisational Cluster

The structures and processes of an organisation can have a substantial influence on the behaviour of individuals [31]. Although, teachers and sports coaches essentially are demonstrating that they are conducting similar practices when teaching a primary PE lesson, the structures and processes behind each of them can be very different. Such as teachers working within one organisation, whereas sports coaches work within several organisations. The sports coaches’ main organisation is that of the coaching company which will have a different vision and role to that of a primary school. For the purpose of the SHARP Principles intervention, both the schools and the coaching company stated that PA in PE lessons should be above 50% MVPA. For the intervention teachers, it appeared that the aim of PE being active whilst also developing skills became the teachers’ main focus, however, for the sports coaches, PA in PE was just one of the many aims of the coaching company. The qualitative data highlighted that the coaching company was a business, and working within the business was the need to suit its customers, hence the coaching company stated that they address many aims in primary PE lessons for instance: physical skills, health, and well-being, holistic development, social skills, etc. Taking this into account, it could be suggested that the mission and goals of the coaching company were not compatible with the main aims of the SHARP Principles intervention, e.g., PE lessons being above >50% MVPA. If the vision of an organisation is not compatible with a health promotion programme then often the intervention will not be effective [31]. Thus, the findings from the SHARP Principles intervention may lead us to question the practice of sports coaches who work across multiple primary school settings and are therefore adapting their PE practice to the needs of the school or ‘customer’. Hence, a coach maybe required to deliver primary PE lessons with different learning intentions for each school in which they work, and therefore active lessons become lost amongst all the other learning intentions. Furthermore, when school staff deliver primary PE lessons, it appears that the appropriate infrastructure is in place to support teachers in being part of the SHARP Principles intervention with a focused aim, however it appears that the sports coaches lacked the necessary support and infrastructure from both the coaching company and the schools in which they were delivering PE. Therefore, a recommendation would be for coaching companies to ensure the appropriate infrastructure is in place, both within the coaching company and the schools in which they are situated to support their sports coaches in the delivery of active PE lessons through having a main vision for PE to be active.

### 4.4. Limitations

All of the participant schools and the coaching company in this research were located in one regional area of England, however the sample included a range of primary school settings and age groups as the aim was to assess the wider implementation of the SHARP Principles intervention. Furthermore, only one coaching company was used in the sample, which may impact upon the generalisability of the findings in relation to the sports coaches. Therefore, future research may test the efficacy of the SHARP intervention when employed across a range of coaching companies. As the research stands, only one post-intervention data collection point took place; therefore, follow up data collection points to test the sustainability of the SHARP Principles intervention would be advised. SOFIT was employed to collect the quantitative data, and although it has been considered as a valid and reliable tool, it may be advisable for future studies to employ accelerometers alongside systematic observation to give further insights and comparisons with similar interventions. Future research could also consider the efficacy of the SHARP Principles when employed in alternative settings, such as children’s sports clubs and special educational needs schools.

## 5. Conclusions

This research provides evidence that the wider implementation of the SHARP Principles intervention was successful when applied by primary school teachers, evidencing mean absolute increases in children’s MVPA of 27.4% in PE lessons, and producing an average MVPA of 74.9% across a range of activity areas. This meets and exceeds the recommended target of >50% MVPA in PE lessons [1]. The SHARP Principles intervention is successful in a primary school setting when applied by teachers as primary schools have the infrastructure and support mechanisms to motivate individual teachers to change their practice. Future recommendations would be for widespread dissemination of the SHARP Principles in primary schools across England to increase children’s MVPA in PE lessons. However, the SHARP Principles intervention was not effective when implemented with sports coaches and it can therefore be concluded that if schools outsource their PE lessons to sports coaches then a number of considerations need to be addressed including: professional development opportunities for individual coaches, support from both the schools in which they work and the coaching company, and one main vision for active PE that would be consistent across all school settings in which they work. Thus, the SHARP Principles are effective when grounded in a supportive organisational culture and it can be concluded that the SHARP Principles intervention is the most effective teaching strategy at increasing MVPA in primary PE when taught by teachers, with increases almost double that of any previously published study internationally. This research has the capacity to influence educational policy and practice internationally.

## Figures and Tables

**Table 1:** Theoretical constructs of the SHARP Principles model (adapted from [24])

**Self Determination Theory** [29]	**Behaviour Change Taxonomy** [30]	**Social Ecological Components** [31]
**Competence**	**Barrier Identification/Problem Solving**	**Individual Level**
Competence developed through knowledge of the SHARP Principles and using the SHARP resource cards.	An initial decision to change behaviour from head teacher/PE coordinator/coaching company leaders and managers. Collection of baseline data provided understanding of the current MVPA levels in primary PE lessons. Discussion between head teacher/PE coordinator/coaching company leaders and managers regarding implementing the SHARP Principles into PE lessons, needs to be grounded in the SHARP Principles model for behaviour change.	Increasing teachers’/coaches’ beliefs e.g. purpose of primary PE linked to ‘active learning time’ MVPA >50%. Developing teachers’/coaches’ pedagogy of PE through a joint planning session; SHARP principles where integrated to increase active learning time. SHARP resource cards were used.
**Relatedness**	**Action Planning**	**Interpersonal Level**
The intervention was supported by the head teacher, PE coordinator, and coaching company leaders and managers which provided an instant support network for the teachers/coaches involved.	Creation of an action plan by Head Teacher/PE Coordinator/coaching company leaders and managers. Targets were set based on the information collected at baseline including children’s MVPA levels during PE. Action planning included: ‘target’, ‘rationale’, ‘action’, ‘timescale’ and ‘evidence/outcome’. Examples of targets where: ‘to increase teachers’ subject knowledge, confidence and planning in primary PE’ and ‘to increase the percentage of active learning time in primary PE to above 50% MVPA through implementation of the SHARP Principles’.	Ongoing support for teachers/coaches from other intervention teachers/coaches, PE Coordinator/head teacher/coaching company leaders and managers. Ongoing reference to the SHARP Principles between school staff/coaching company staff.
**Autonomy**	**Provide Instruction on How to Perform the Behaviour**	**Organisational Level**
Teachers/coaches to be in control of their own behaviour. Teachers/coaches chose the content of the lesson and planned the sessions independently, the SHARP Principles were a layer that could be applied to any lesson content.	Providing instruction, involved ‘telling’ the teachers/coaches ‘how’ to perform the behaviour. In this instance, joint planning sessions (30 min) took place with year group teachers and the lead researcher. In the planning sessions there was a focus on the integration of the SHARP principles to increase children’s active learning time to above 50% MVPA. The SHARP resource cards were shared.	Ongoing support from the head teacher/coaching company leaders and directors. Integrating the SHARP Principle into PE/PA policy.

**Table 2:** SHARP Principles: increasing active learning time in primary PE [24].

Stretching whilst moving	During the warm-up section of a PE lesson, activities are to include dynamic movements and stretches, replacing the traditional static stretching routines [32]. Dynamic movements should be designed to elevate and maintain a higher core body temperature, whilst also engaging children in a fun, active, and purposeful warm-up. A dynamic warm-up includes various movements that engage the lower and upper body [33]. A dynamic warm-up assists in increasing children’s MVPA and could therefore allow for greater explosive effort during subsequent activities [34]. Examples of dynamic stretches include: side shuffles, jump and twist, high knees, heel flicks, jumping jacks and skipping [33]. The teacher must ensure that the dynamic movements will prepare the children for the activities that will follow in the skill development and then application of those skills.		
High repetition of motor skills	This principle is based on the notion that children cannot become physically skilled if they are not engaged in active learning [35]. In order to increase active learning time, teachers must ensure that each child has the opportunity to engage in the task at hand. For instance: reducing/eliminating queues so that children are not waiting their turn; having small sided games or group work such as 3 vs. 3 (which will increase the amount of times children have to apply an acquired skill and help to eliminate children being on the peripheral of, or excluded from a game/activity); and increasing the amount of equipment available to the children and/or increasing the number of stations.		
Accessibility through differentiation	All children should be set tasks that are appropriate to their physical, cognitive, and social development, which will enable them to engage in active learning time. Teachers should ensure that they are familiar with the STEP framework (Space, Task, Equipment, and People) for effective differentiation of activities [36]. An example of the acronym STEP for a gymnastics lesson would be:		
	STEP	Easier	Harder
	Space	Working in their own space	Sharing multiple stations with others
	Task	Reducing the number of elements to be included in a sequence	Increasing the number of elements to be included in a sequence
	Equipment	Using the floor and mats	Using the floor, mats, and apparatus
	People	Working with a partner	Working in a small group
Reducing sitting and standing	As PE is the only required curriculum subject to provide MVPA to all children [37]; this principle aims to develop teachers’ awareness of the amount of time children are sitting and standing during the lesson in relation to knowledge transfer, teacher feedback and organisation of equipment (similar to the SPARK PE programme which placed an emphasis on efficient teacher feedback, whilst the child remained on task [21]. Examples of this principle include: When a teacher is providing feedback or questioning learners, often they do not need to stop the whole class, instead they can just target and stop a group of learners or an individual child. Engaging children in activity as soon as possible at the start of the lesson through concise questioning and feedback. Ensuring equipment is ready, organized, and accessible at the start and throughout the lesson.		
Promoting in-class physical activity	If teachers are to assist in the development of children’s lifelong PA, they must make a conscious effort to change their instruction behaviours during PE lessons promoting in class PA [37]. This principle is also linked to the assessment of PA during PE lessons using the SOFIT observational tool [38]. An example of the promotion of in-class PA includes ‘great team work’, ‘keep moving’, and ‘look for space’.		
